# Sleep Patterns and Myopia Among School-Aged Children in Singapore

**DOI:** 10.3389/fpubh.2022.828298

**Published:** 2022-03-25

**Authors:** Mijie Li, Chuen-Seng Tan, Lingqian Xu, Li-Lian Foo, Fabian Yap, Chen-Hsin Sun, Elaine K. H. Tham, Shirong Cai, Marcus Ang, Seang-Mei Saw, Charumathi Sabanayagam

**Affiliations:** ^1^Saw Swee Hock School of Public Health, National University of Singapore, Singapore, Singapore; ^2^Singapore Eye Research Institute, Singapore National Eye Centre, Singapore, Singapore; ^3^Ophthalmology and Visual Sciences Academic Clinical Programme, Duke-NUS (National University of Singapore) Medical School, Singapore, Singapore; ^4^Department of Maternal Fetal Medicine, KK Women's and Children's Hospital, Singapore, Singapore; ^5^Yong Loo Lin School of Medicine, National University of Singapore and National University Health System, Singapore, Singapore; ^6^Singapore Institute for Clinical Sciences, Agency for Science, Technology and Research (A^*^STAR), Singapore, Singapore

**Keywords:** myopia, sleep, refractive error, axial length, children

## Abstract

**Purpose:**

To evaluate the associations of sleep factors with myopia, spherical equivalent (SE), and axial length (AL) in elementary school-aged children from the Growing Up in Singapore Towards healthy Outcomes (GUSTO) birth cohort.

**Methods:**

This cross-sectional study included multi-ethnic children who participated in the GUSTO prospective birth cohort and were delivered in two major tertiary hospitals in Singapore (2009–2010). Sleep factors and myopia outcomes were assessed at the 8- and 9-year study visits, respectively. Parent-reported sleep quality was assessed with the Children's Sleep Habits Questionnaire (CSHQ) total scores. Additionally, each child's sleep duration, timing (bedtime; waketime), and the consistency of sleep duration or timing (i.e., the difference between weekends and weekdays) were parent-reported. Outcomes included cycloplegic SE, myopia (SE ≤ −0.5 D) and AL. Eye measurements from both eyes were included in the analyses. Multivariable linear or logistic regression with Generalized Estimating Equations were used to account for the correlation between paired eyes and confounders in the associations of sleep factors at age 8 and myopia at age 9.

**Results:**

A total of 572 multi-ethnic children (49.5% boys; 56.1% Chinese) aged 9 years were included in the analyses. Overall, 37.3% of eyes were myopic. Children reported a mean total CSHQ score of 46 [standard deviation (*SD*) = 6]. The mean duration of sleep was 9.2 (*SD* = 1.0) hours per day (h/day), with 59.9% of children reporting sufficient sleep (≥9 h/day) based on guidelines recommended by the National Sleep Foundation, USA. The mean bedtime and wake time were 22:00 (*SD* = 00:53) and 07:08 (*SD* = 00:55), respectively. In multivariable regression models, total CSHQ scores, the duration of sleep, bedtime and wake time were not significantly associated with myopia, SE, or AL (*p* ≥ 0.05 for all), adjusting for gender, ethnicity, time outdoors, near-work, parental myopia, maternal education levels (and additionally the child's height when the outcome was AL). Similarly, the consistency of both the duration and timing of sleep (across weekends and weekdays) were not significantly associated with myopia, SE, or AL (*p* ≥ 0.05 for all).

**Conclusion:**

In this cross-sectional study, sleep quality, duration, timing, and the consistency of specific sleep factors were not independently associated with myopia, SE, or AL among elementary school-aged children in Singapore. Large longitudinal studies are warranted to corroborate these results.

## Introduction

Myopia has reached epidemic levels in urban East Asia and Singapore, affecting up to 80–90% of young adults (1, 2). The onset of myopia in childhood increases the risks of high myopia (3), and consequently, myopic macular degeneration (4), cataracts and glaucoma (5) in adulthood. Genetic factors and environmental factors [notably increased education (6) and decreased time outdoors (7–10)] are associated with myopia onset, and myopic children are more likely to engage in near work (11). However, these factors only partially accounted for the risk of myopia (12, 13), and other environmental factors may be involved (14).

The sleep-wake cycle is closely related to the circadian system (15). A recent meta-analysis of genome-wide association studies has linked genetic factors regulating circadian rhythms to refractive error development (16). While sleep patterns in children are closely linked to lifestyle behavioral factors (17), sleep disruptions may also result from (18) or result in (19) perturbations to circadian rhythms. Thus, the evaluation of sleep factors may offer insights into potential circadian effects on myopia. Several cross-sectional and prospective studies have evaluated the association between sleep and myopia, but the findings are mixed. In cross-sectional studies, although a lower quality of sleep (20), shorter (21, 22) or longer (23) duration of sleep, and later bedtime (24) were associated with higher odds of myopia in some studies, null associations were reported in other studies with regards to the quality (25) or duration (20, 24, 26) of sleep and, bedtime (26). Importantly, only a few studies have been conducted with cycloplegic refraction data (20, 21, 25). Two prospective studies with cycloplegic data reported mixed findings between a limited set of sleep factors (duration of sleep or bedtime) and myopia (27, 28). In the study by Wei et al., neither duration of sleep nor bedtime was associated with 4-year incidence of myopia (*p* ≥ 0.05 for all) among 1,887 Chinese children aged 5–9 years at baseline (27). Conversely, in another study by Liu et al., late bedtime, but not the duration of sleep (*p* ≥ 0.05), was associated with 2-year myopia incidence {odds ratio (OR) = 1.45, 95% confidence interval (CI) [1.05, 2.00], *p* = 0.02} in 4,982 Chinese children aged 6–9 years (at baseline) participating in a school-based outdoor trial (28). Moreover, there is a lack of evidence on the effects of the quality (20, 25) or consistency of sleep [linked to sleep problems (29) or circadian phase shifts (30)] on myopia. Overall, given the scarcity of studies with cycloplegic refraction data and the limited range of sleep factors studied, associations between sleep factors and myopia remain poorly understood.

We aim to evaluate the associations of sleep factors (quality, duration, timing, and consistency) with myopia in school-aged children from the Growing Up in Singapore Towards healthy Outcomes (GUSTO) birth cohort.

## Methods

### Study Population

The Singapore GUSTO birth cohort recruited pregnant mothers and babies born to these mothers from two major public maternity hospitals in Singapore (National University Hospital and KK Women's and Children's Hospital) between 2009 and 2010 (31). The children in the GUSTO birth cohort were followed up prospectively to assess multiple childhood outcomes at various study visits. Data for this study was derived from children who attended both the 8-year study visit (point of sleep exposure assessments) and the 9-year study visit (point of ocular outcomes assessments as part of the GUSTO myopia study). Of the 1,176 children at birth, 716 (61%) returned at the 9-year GUSTO myopia study visit for ocular examinations. Of these 716 children, 709 who were not on myopia control treatment were eligible. Among those eligible, 572 children with both cycloplegic refraction outcomes and sleep exposure data were included in the final analyses [137 children without available cycloplegic refraction data (*n* = 82) or CSHQ questionnaires (*n* = 55) were excluded]. The majority of children in Singapore (over 90%) (32) attend compulsory education in government elementary schools, which have similar start times (around 7:30 a.m.) (33). Parents and children provided written consent and assent before participation. Ethics approval was obtained from the National Healthcare Group Domain Specific Review Board (D/2009/021), the Singhealth Centralized Institutional Review Board (2018/2767) and both Review Boards (2018/2270; R1517/16/2018), respectively. The conduct of this study adhered to the tenets of the Declaration of Helsinki.

### Sleep Factors (Exposures)

The Children's Sleep Habits Questionnaire (CSHQ) (parent-reported) was administered for the first time at the 8-year visit in the GUSTO birth cohort. The CSHQ has been widely used to assess sleep patterns and to screen for sleep problems in children aged 4–10 years across various ethnic groups (34–36). Validation studies for the CSHQ have been conducted in community samples across multiple countries [including the United States of America (34, 37), China (38), Portugal (39), Germany (40), and Italy (41)], with adequate full-scale internal consistency (given by Cronbach's alpha) ranging between 0.68 and 0.82. The quality of sleep was assessed by the total CSHQ score, calculated from the sum of 8 CSHQ subscales encompassing the major presenting sleep complaints in children (bedtime resistance, sleep onset delay, sleep duration, sleep anxiety, night wakings, parasomnias, sleep-disordered breathing, and daytime sleepiness). A higher total CSHQ score indicates a lower quality of sleep (or more sleep problems).

In addition to the CSHQ questionnaire, parents also responded to additional questions on other sleep factors including the duration [duration of sleep, duration in bed (night only, naps only, or total combining the sum of night and naps)], timing (bedtime, wake time), and consistency of sleep. Parents reported the duration of sleep based on the following question: “In the past week or most recent typical week, what is the child's usual amount of sleep each day combining nighttime sleep and naps?”. Duration in bed during the night (or during naps) was calculated as the interval between the child's bedtime and wake time in the morning (or between usual naptime and time of the day when the child wakes after the nap). All parents completed the electronic questionnaires in quiet and private settings. The daily duration of sleep, hours per day (h/day), or the duration spent in bed (h/day) across all days of the week were computed as follows: 5/7 × daily hours on weekdays (h/day) + 2/7 × daily hours on weekends (h/day). Similar calculations were performed in the computation of daily bedtime and wake time (clock hours) across all days of the week. The consistency of the duration and timing of each sleep factor was computed as the difference in reported values between weekends [Saturday and Sunday (WE)] and weekdays [Monday to Friday (WD)] for each child [i.e., WE-WD; (42)].

### Ocular Examination (Outcomes)

Cycloplegic spherical equivalent (SE) and AL were assessed using autorefractors (Canon RK-5/RK-F2, Canon; Japan) and optical biometers (IOL Master 500, Carl Zeiss-Meditec; Germany), respectively, at the 9-year visit. Cycloplegia was induced using 3 drops of 1% cyclopentolate hydrochloride, instilled 5 minutes apart. Autorefraction was performed at least 30 min after the first drop, with pupil dilation of ≥6 mm. SE was calculated as the sphere power plus half of the cylinder power. The main refractive error outcomes were myopia, SE, and AL. In the current study, myopia was defined as SE ≤ −0.5 D. Emmetropia was defined as SE > −0.5 D to SE < 2.0 D, hyperopia was defined as SE ≥ 2.0 D, and astigmatism was defined as cylinder power >0.75 D.

### Anthropometric and Questionnaire Measurements

Paper questionnaires were administered to parents to collect demographic information and information on other potential confounders (43). Parents reported on their child's gender, ethnicity (Chinese or non-Chinese comprising Malays, Indians, and others) and the daily duration (h/day) spent on time outdoors (including physical and leisure activities) or near-work activities (i.e., reading, writing, drawing, crafts, use of computers, or hand-held devices), on both WE and WD, in the past month, at the 9-year visit. Similarly, the daily duration of time outdoors or near-work activities across all days of the week (h/day) was computed as follows: 5/7 × daily hours on WD (h/day) + 2/7 × daily hours on WE (h/day). As standing height may be associated with axial length (44), each child's standing height [centimeters (cm)] was measured using stadiometers (Seca 213, Seca, Hamburg, Germany). Additionally, we collected parent-reported information on maternal education levels (secondary school and lower or GCE O levels and above) (45, 46) and the number of myopic parents of the child (none or at least one parent) (47, 48), as these factors have been associated with myopia. All parents completed the paper questionnaires in quiet and private settings.

### Statistical Analysis

All sleep factors (exposures) were analyzed as both continuous and categorical variables. Children with a duration of sleep of ≥9 h/day were considered to have met the recommendations for sufficient sleep, based on guidelines for school-aged children aged 6–13 years (49). Given that recommended clinical cutoffs for other sleep factors are lacking (50), sleep quality, duration in bed and timings of sleep were assessed as tertile categories. The consistency of sleep factors was also assessed as binary categories, where differences between WE and WD that were within an hour [approximate median and referencing previously cited cut-offs (51)] corresponded to the group with higher consistency. Sensitivity analyses were conducted by assessing the consistency of sleep factors as the absolute difference between WE and WD. Myopia was analyzed as a binary variable whereas outcomes SE and AL were analyzed as continuous variables.

Two-sample *t*-tests and Fisher's exact tests were used to compare continuous and categorical characteristics of children included in and excluded from the analyses, respectively. Paired *t*-tests and McNemar's tests were used to compare continuous and binary variables across WE and WD. In the analyses of ocular measures and tests of associations between sleep factors and each outcome after 1 year, eye measurements from each child were analyzed. In the tests of associations, multivariable logistic (myopia) or linear (SE or AL) regression models with Generalized Estimating Equations (GEE) (52, 53) were used to account for the correlation between paired eyes and confounders. Confounders considered in an initial multivariable model were based on a priori knowledge from the literature and included gender (54, 55), ethnicity (56, 57), near-work (58, 59), time outdoors (7, 8), the number of myopic parents (47, 48), and maternal education level (45, 46) [additionally child's height in models with AL (44) as the outcome]. Final multivariable models were determined with backward manual stepwise selection, starting with the full model and identifying a single confounder with the least significant *p*-value (if *p* > 0.05) to exit the model at each step. Instead of an automated procedure, manual exclusion and inclusion of confounders were made as the decision for the final model accounted for both model fits (e.g., quasi-likelihood under the independence model criterion, QICu) and strong evidence from the literature. Wald tests were conducted to test for any association between the tertile categories and each outcome, while tests of linear trends were performed by modeling tertile categories of specific sleep factors as numeric variables (first to third tertiles were assigned numerical values 1–3). All statistical tests were two-sided with statistical significance set at *p* < 0.05. Estimated measures of association and their 95% confidence intervals (CI) were reported. Statistical analyses were performed using Stata v13 (StataCorp, USA).

## Results

**Figure 1:**
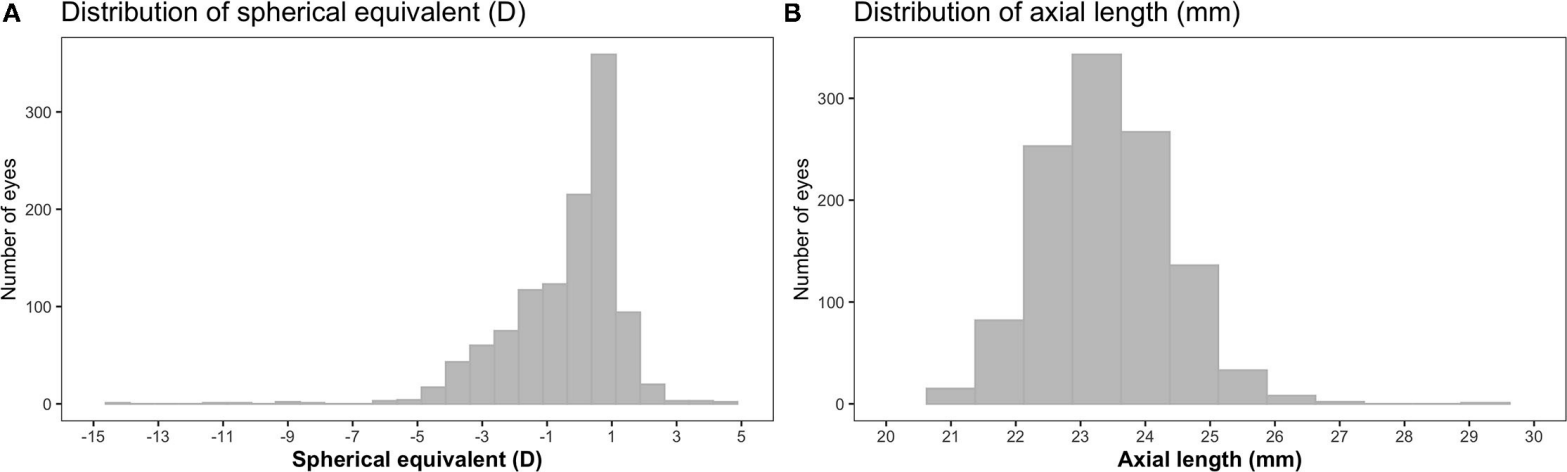
Distributions of spherical equivalent (SE) **(A)** and axial length (AL) **(B)** in children from the Growing Up in Singapore Towards healthy Outcomes (GUSTO) cohort (*n* = 572). D, diopters.

A total of 572 children (1,144 eyes) were included in the analyses, of which 283 (49.5%) were boys and 321 (56.1%) were Chinese. The majority of the children had at least one myopic parent (76.7%) or mothers with higher education levels (67.3%). On average, children reported a mean duration of time outdoors of 1.7 [standard deviation (*SD*) = 1.6] h/day and near work duration of 5.5 (*SD* = 3.0) h/day. There were 427 myopic eyes (37.3%), 689 emmetropic eyes (60.3%) and 28 hyperopic eyes (2.4%) (there were no eyes with astigmatism). The mean of SE was −0.4 (*SD* = 1.7) D and the mean of AL was 23.4 (*SD* = 1.0) mm (Figure 1). The mean AL of eyes demonstrated an increasing trend with increasing severity of myopic SE (*p*-linear trend <0.001): 23.0 (*SD* = 0.7) mm [SE > −0.50 D], 23.9 (*SD* = 0.7) mm [SE ≤ −0.50 D to SE > −3.0 D], 24.7 (*SD* = 0.7) mm [SE ≤ −3.0 D to SE > −5.0 D], and 26.2 (*SD* = 1.1) mm [SE ≤ −5.0 D]. Being Chinese (compared to non-Chinese), spending less time outdoors or having at least one myopic parent (compared to no myopic parent) was associated with higher odds of myopia and more myopic SE (*p* <0.05 for all). Similarly, being Chinese, female, taller, or having at least one myopic parent was associated with longer AL (*p* < 0.05 for all). Comparing eligible children included in (*n* = 572) and excluded (*n* = 137) from (due to the lack of cycloplegic refraction data or CSHQ) analyses, there were no differences in the proportion of myopic eyes, mean of SE and mean of AL (*p* > 0.05 for all). Additionally, children included for analyses had comparable proportions of boys or Chinese, and comparable proportions of children with at least one myopic parent or mothers having higher educational levels (*p* > 0.05 for all), compared to those excluded. Furthermore, children included (compared to excluded) for analyses did not differ in the mean of the duration of time outdoors or height (*p* > 0.05 for all), but had higher levels of near-work [5.5 (*SD* = 3.0) vs. 4.8 (*SD* = 2.6) h/day, *p* = 0.010].

Across all days, the mean duration of sleep was 9.2 (*SD* = 1.0) h/day (range: 4.3–13.1 h/day), with 59.9% of children meeting the recommendations for sleep sufficiency (Table 1). The mean of total CSHQ score was 45.8 (*SD* = 6.2), with mean of subscale scores of 9.7 (*SD* = 2.6) (bedtime resistance), 1.3 (*SD* = 0.5) (sleep onset delay), 4.0 (*SD* = 1.3) (sleep duration), 6.4 (*SD* = 2.1) (sleep anxiety), 3.6 (*SD* = 0.9) (night wakings), 8.1 (*SD* = 1.2) (parasomnias), 3.3 (*SD* = 0.6) (sleep-disordered breathing), and 13.1 (*SD* = 3.0) (daytime sleepiness). On weekends, children reported a significantly longer duration of sleep (with a higher proportion of children achieving sleep sufficiency), longer duration in bed at night, shorter duration in bed during naps, later bedtime and wake time, compared to weekdays (*p* < 0.001 for all).

**Table 1:** Summary of sleep patterns and Children's Sleep Habits Questionnaire (CSHQ) scores in children from the Growing Up in Singapore Towards healthy Outcomes (GUSTO) cohort (*n* = 572).

	**Mean (*****SD*****) or** ***n*** **(%)**^**+**^			
**Sleep factors**	**All days^†^**	**Weekends**	**Weekdays**	**p^‡^**
CSHQ total scores, mean (*SD*)	45.8 (6.2)			
Duration of sleep [hours/day, (h/day)], mean (*SD*)	9.2 (1.0)	9.8 (1.2)	8.9 (1.1)	<0.001
Sleep sufficiency^#^, *n* (%)				<0.001
Meeting recommendations (≥9 h/day)	340 (59.9)	487 (85.7)	328 (57.4)	
Below recommendations (<9 h/day)	228 (40.1)	81 (14.3)	243 (42.6)	
Duration in bed (h/day), mean (*SD*)				
Total (night and naps) (h/day)	9.5 (1.0)	10.1 (1.2)	9.3 (1.1)	<0.001
Night only (h/day)	9.1 (0.9)	9.8 (1.0)	8.9 (1.0)	<0.001
Naps only (h/day)	0.4 (0.6)	0.3 (0.7)	0.4 (0.8)	<0.001
Bedtime (clock hours), mean (*SD*)	21:59 (00:53)	22:34 (1:00)	21:45 (00:54)	<0.001
Wake time (clock hours), mean (*SD*)	07:08 (00:55)	08:24 (01:14)	06:38 (1:01)	<0.001
Consistency of duration of sleep^§^ (h), mean (*SD*)	0.9 (1.2)	–	–	
Consistency of duration in bed^§^ (h), mean (*SD*)				
Total (night and naps) (h)	0.8 (1.3)	–	–	
Night only (h)	0.9 (1.2)	–	–	
Naps only (h)	−0.1 (0.8)	–	–	
Consistency of bedtime^§^ (clock hours), mean (*SD*)	00:49 (00:40)	–	–	
Consistency of wake time^§^ (clock hours), mean (*SD*)	01:46 (01:17)	–	–	

*SD, standard deviation; %, percentage; CSHQ, Children's Sleep Habits Questionnaire*.

† Values presented for sleep duration and timing are mean daily values across all days of the week (h/day), aggregated using the following formula: [5/7* 100 daily hours on weekdays (h/day) + 2/7* 100 daily hours on weekends (h/day)].

‡ *p-values from two-sample paired t-tests (continuous variables) or McNemar's test (categorical variables) indicate if there are any significant differences comparing weekends and weekdays for each sleep factor*.

§ *Consistency of each sleep factor was defined as the difference in values between weekends and weekdays (i.e., weekends-weekdays)*.

# *Missing sleep sufficiency data for all days (n = 4), weekends (n = 4), and weekdays (n = 1)*.

+ *Continuous variables were presented as mean (SD) and categorical as n (%)*.

In multivariable models, there were no significant associations between total CSHQ scores, duration of sleep, duration in bed (total, night only, or naps only) or timings of sleep (bedtime, wake time) and myopia, SE, or AL (*p* ≥ 0.05 for all), adjusting for gender, ethnicity, near-work, time outdoors, parental myopia, and maternal education (additionally child's height when the outcome was AL) (Table 2). Comparing children meeting and below the recommendations for sufficient sleep, there were no significant differences in myopia, SE, or AL (*p* ≥ 0.05 for all). Similarly, when sleep factors were analyzed as tertile categories, the Wald tests (*p* ≥ 0.05 for all) and tests of linear trend (*p*-linear trend ≥ 0.05 for all) suggested no significant associations with myopia, SE, or AL.

**Table 2:** Associations of sleep factors with myopia (SE ≤ −0.5 D), spherical equivalent (D) and axial length (mm) in children from the Growing Up in Singapore Towards healthy Outcomes (GUSTO) cohort (*n* = 572).

		**Myopia (SE** **≤−0.5 D)**				**SE (D)**				**AL (mm)**			
		**Univariable** **model**		**Multivariable** **model**^**†**^		**Univariable** **model**		**Multivariable** **model**^**†**^		**Univariable** **model**		**Multivariable** **model**^**†**^	
**Sleep factors**	**Eyes^#^**	**OR** **[95% CI]**	***p*** ^**‡**^	**OR** **[95% CI]**	***p*** ^**‡**^	**Beta coefficient** **[95%CI]**	***p*** ^**§**^	**Beta coefficient** **[95%CI]**	***p*** ^**§**^	**Beta coefficient** **[95%CI]**	***p*** ^**§**^	**Beta coefficient** **[95%CI]**	***p*** ^**§**^
Total CSHQ score (per point increment)	1,006	0.99 [0.96, 1.01]	0.34	0.99 [0.96, 1.02]	0.38	−0.001 [−0.03, 0.02]	0.93	−0.0004 [−0.03, 0.03]	0.97	−0.003 [−0.02, 0.01]	0.64	−0.01 [−0.02, 0.01]	0.37
Duration of sleep^+^ [hours/day, (h/day)] (per hourly increment)	1,044	1.16 [0.99, 1.38]	0.071	1.15 [0.95, 1.39]	0.16	−0.06 [−0.20, 0.07]	0.37	−0.04 [−0.18, 0.10]	0.59	−0.003 [−0.08, 0.08]	0.95	0.02 [−0.06, 0.09]	0.64
Sleep sufficiency^+^									
≥9 h/day	623	(Ref)		(Ref)		(Ref)		(Ref)		(Ref)		(Ref)	
<9 h/day	421	0.87 [0.62, 1.21]	0.41	0.89 [0.62, 1.28]	0.53	0.01^¶^ [−0.28, 0.30]	0.96	−0.02^¶^ [−0.32, 0.28]	0.88	0.07^¶^ [−0.09, 0.24]	0.40	0.03^¶^ [−0.13, 0.18]	0.75
Duration in bed^+^ (h/day)													
Total (h/day) (per hourly increment)	1,020	1.01 [0.85, 1.20]	0.90	1.001 [0.82, 1.22]	0.99	0.04 [−0.10, 0.17]	0.62	0.02 [−0.12, 0.17]	0.75	−0.03 [−0.12, 0.05]	0.46	−0.001 [−0.08, 0.08]	0.97
Night only (h/day) (per hourly increment)	1,044	1.14 [0.95, 1.38]	0.16	1.11 [0.90, 1.37]	0.33	−0.07 [−0.22, 0.08]	0.34	−0.06 [−0.21, 0.10]	0.45	−0.003 [−0.10, 0.09]	0.96	0.02 [−0.07, 0.11]	0.68
Naps only (h/day) (per hourly increment)	1,022	0.78 [0.59, 1.02]	0.072	0.82 [0.61, 1.10]	0.18	0.21 [0.02, 0.41]	0.033	0.16 [−0.04, 0.37]	0.12	−0.06 [−0.18, 0.06]	0.30	−0.03 [−0.15, 0.08]	0.55
Bedtime^+^ (per clock hour increment)	1,044	0.92 [0.76, 1.12]	0.41	0.87 [0.71, 1.07]	0.19	0.11 [−0.04, 0.26]	0.14	0.14 [−0.01, 0.29]	0.074	−0.04 [−0.13, 0.05]	0.37	−0.03 [−0.12, 0.06]	0.49
Wake time^+^ (per clock hour increment)	1,046	1.05 [0.88, 1.26]	0.58	0.97 [0.78, 1.21]	0.78	0.04 [−0.11, 0.18]	0.61	0.08 [−0.09, 0.25]	0.36	−0.04 [−0.13, 0.05]	0.37	−0.01 [−0.10, 0.08]	0.80

*SD, standard deviation; SE, spherical equivalent; D, diopters; AL, axial length; OR, odds ratio; CI, confidence interval; CSHQ, Children's Sleep Habits Questionnaire*.

† *Multivariable models adjusted for gender, ethnicity, near-work, time outdoors, parental myopia and maternal education. When the outcome was AL, multivariable models further adjusted for the child's height*.

‡ *p-values from logistic regression with generalized estimating equations (GEE)*.

§ *p-values from linear regression with GEE*.

# *Number of eyes in multivariable models for outcome AL, representing the minimum number of eyes analyzed are presented. The number of eyes analyzed for all univariable (for outcomes myopia, SE, and AL) and multivariable models (for outcomes myopia and SE) are equal to or larger than the values presented*.

+ Values presented are mean daily values across all days of the week (h/day), aggregated using the following formula: [5/7* 100 daily hours on weekdays (h/day) + 2/7* 100 daily hours on weekends (h/day)].

¶ *The mean difference was presented for sleep sufficiency*.

In multivariable models, there were similarly no significant associations between the consistency of sleep factors (difference between WE and WD), in terms of duration of sleep, duration in bed (total, night, or nap only), bedtime or wake time and myopia, SE, or AL (*p* ≥ 0.05 for all), adjusting for gender, ethnicity, near-work, time outdoors, parental myopia, and maternal education (additionally child's height when the outcome was AL) (Table 3). Children with lower, compared to higher consistency of sleep factors did not differ significantly in myopia, SE, or AL outcomes (*p* ≥ 0.05 for all). In sensitivity analyses, there was similarly no significant associations between the consistency of sleep factors (absolute difference in reported values between WE and WD) and myopia, SE, or AL (*p* ≥ 0.05 for all).

**Table 3:** Associations of the consistency of sleep duration and timing with myopia (SE ≤ −0.5 D), spherical equivalent (D) and axial length (mm) in children from the Growing Up in Singapore Towards healthy Outcomes (GUSTO) cohort (*n* = 572).

		**Myopia (SE** **≤−0.5 D)**				**SE (D)**				**AL (mm)**			
		**Univariable** **model**		**Multivariable** **model**^**†**^		**Univariable** **model**		**Multivariable** **model**^**†**^		**Univariable** **model**		**Multivariable** **model**^**†**^	
**Sleep factors^**#**^**	**Eyes^+^**	**OR** **[95% CI]**	***p*** ^**‡**^	**OR** **[95% CI]**	***p*** ^**‡**^	**Beta coefficient** **[95%CI]**	***p*** ^**§**^	**Beta coefficient** **[95%CI]**	***p*** ^**§**^	**Beta coefficient** **[95%CI]**	***p*** ^**§**^	**Beta coefficient** **[95%CI]**	***p*** ^**§**^
Consistency of duration of sleep [hours, (h)] (per hourly increment)	1,044	0.91 [0.80, 1.04]	0.17	0.91 [0.78, 1.05]	0.20	0.06 [−0.06, 0.17]	0.33	0.04 [−0.08, 0.15]	0.52	−0.03 [−0.09, 0.04]	0.46	0.003 [−0.06, 0.07]	0.93
Consistency of duration in bed (h)													
Total (night + nap) (h) (per hourly increment)	1,020	0.93 [0.82, 1.05]	0.24	0.90 [0.79, 1.03]	0.14	0.005 [−0.11, 0.12]	0.93	0.02 [−0.09, 0.14]	0.71	0.003 [−0.06, 0.07]	0.93	−0.003 [−0.06, 0.06]	0.93
Night only (h) (per hourly increment)	1,044	0.95 [0.83, 1.08]	0.43	0.95 [0.82, 1.10]	0.50	0.02 [−0.09, 0.14]	0.68	0.01 [−0.10, 0.13]	0.81	−0.03 [−0.10, 0.03]	0.32	−0.02 [−0.08, 0.04]	0.52
Naps only (h) (per hourly increment)	1,022	0.88 [0.73, 1.06]	0.18	0.83 [0.67, 1.03]	0.093	0.004 [−0.15, 0.15]	0.96	0.04 [−0.12, 0.19]	0.65	0.04 [−0.04, 0.13]	0.32	0.02 [−0.06, 0.10]	0.58
Consistency of bedtime (per clock hour increment)	1,044	0.86 [0.67, 1.10]	0.23	1.001 [0.76, 1.33]	1.00	0.07 [−0.12, 0.27]	0.47	−0.05 [−0.27, 0.17]	0.65	−0.07 [−0.20, 0.06]	0.28	0.02 [−0.10, 0.14]	0.75
Consistency of wake time (per clock hour increment)	1,046	0.92 [0.81, 1.04]	0.17	0.96 [0.84, 1.09]	0.53	0.04 [−0.05, 0.13]	0.39	−0.001 [−0.09, 0.09]	0.98	−0.05 [−0.11, 0.01]	0.11	−0.01 [−0.07, 0.04]	0.66

*SD, standard deviation; SE, spherical equivalent; D, diopters; AL, axial length; OR, odds ratio; CI, confidence interval; CSHQ, Children's Sleep Habits Questionnaire*.

† *Multivariable models adjusted for gender, ethnicity, near-work, time outdoors, parental myopia and maternal education. When the outcome was AL, multivariable models further adjusted for the child's height*.

‡ *p-values from logistic regression with generalized estimating equations (GEE)*.

§ *p-values from linear regression with GEE*.

# *Consistency of sleep factors was defined as the difference between reported values for weekends and weekdays*.

+ *Number of eyes from multivariable models for outcome AL, representing the minimum number of eyes analyzed are presented. The number of eyes analyzed for all univariable (for outcomes myopia, SE, and AL) and multivariable models (for outcomes myopia and SE) are equal to or larger than the values presented*.

## Discussion

In this cross-sectional study, sleep quality, duration, timing, and the consistency of specific sleep factors were not independently associated with myopia, cycloplegic SE or AL among school-aged children in Singapore.

In this study, the mean total CSHQ score was comparable to other studies on children of a similar age living in China (44) (reference number 20) or Australia (AUS) (44–54) (reference number 60). Compared to the mean duration of sleep in this study (9.2 h/day), other studies have reported mean durations of 9.5 h/day (China) (28), 10 h/day (Australia) (60), and 10.2 h/day (United States of America, USA) (61). We reported relatively later mean bedtime (21:59), compared to Chinese (21:02) (61), Australian (21:00) (60), or American children (20:27) (61), but children in this study do not stand out in mean wake time (60–62). Differences in sleep factors across studies may reflect varied educational loads (higher in Asian countries) (63–65), sleep practices (61), social schedules, or other lifestyle behaviors (17).

The overall null associations between sleep factors and myopia, SE, or AL in this study concur with the lack of associations between specific sleep factors and myopia reported in other prospective studies (27, 28, 66). Our findings corroborate with the 4-year prospective study by Wei et al., where duration of sleep and bedtime were not significantly associated with myopia incidence, myopic progression, or AL elongation [*p* ≥ 0.05 for all; (27)]. Similarly, in the 2-year prospective study by Liu et al., duration of sleep was not significantly associated with myopia incidence [*p* ≥ 0.05; (28)]. Moreover, in a previous report from the same GUSTO prospective study, we reported no significant associations between the duration of sleep or number of night wakings at 12 months and myopia, SE, and AL in 376 children aged 3 years [*p* ≥ 0.05 for all; (66)]. Additionally, in a cross-sectional study of 474 pairs of Chinese children aged 13–14 years, sleep problems (total CSHQ scores) were also not found to be significantly associated with myopia [*p* ≥ 0.05; (25)].

Conversely, significant associations between sleep factors and myopia have been reported in the Liu et al. study and in two other cross-sectional studies (20, 21, 28). In the Liu et al. study, later bedtime (defined as ≥9:30 p.m. vs. <9 p.m.), but not the duration of sleep, was associated with higher 2-year myopia incidence (OR = 1.45, 95% CI [1.05, 2.00], *p* = 0.02), adjusting for age, gender, residency area (urban/suburban) and outdoor intervention group (28). Of note, children with later bedtime in Liu et al. also reported significantly less time outdoors, more near-work, and a higher likelihood of having more myopic and educated parents (28), all of which may be associated with more myopia (7, 43, 45, 48, 58). On the other hand, consistent with the 4-year prospective study by Wei et al., the current study found no significant associations (*p* > 0.05 for all), but there were suggestions of an inverse trend where those with a later bedtime had lower odds of myopia, less myopic SE and shorter AL. In a cross-sectional study of 15,316 Chinese students aged 6–18 years by Xu et al., shorter duration of sleep (<7 h/day vs. ≥9 h/day) was associated with higher odds of myopia [OR = 3.37, 95% CI [3.07–3.70], *p* < 0.001; (21)]. However, daily time outdoors, a key risk factor for myopia, was not accounted for in the Xu et al. study. Conversely, in the current study, both a higher duration of sleep and a higher duration spent in bed at night were not significantly associated with myopia outcomes (*p* > 0.05 for all), and there were suggestions of a positive trend between these factors and myopia, myopic SE or AL. These null results corroborate the null findings from three other prospective studies (27, 28, 66). In another cross-sectional study of 1902 Chinese children aged 6–12 years, by Zhou et al., higher total CSHQ scores (or lower quality of sleep) was associated with higher odds of myopia [OR = 1.01, 95% CI [1.00, 1.02], *p* = 0.014; (20)]. Similar to the small magnitude of estimates reported by Zhou et al., the estimates between CSHQ scores and myopia outcomes were close to null in the current study, with no significant associations or trends (*p* > 0.05 for all). Additionally, despite accounting for time outdoors, adjustments were not made for parental myopia in the Chinese study. Similar to previous studies (7, 9, 48, 67), the current study showed that higher time outdoors had an inverse association, while having myopic parents had a positive association with myopia. Thus, although significant associations may arise from differences in population characteristics, sample sizes or sleep assessment instruments, these findings have been inconsistent overall and require careful interpretation, given residual confounding by known risk factors of myopia could not be ruled out.

Overall, the results in this study suggest that sleep factors may not be independently associated with myopia. The inconsistent evidence overall, together with the null associations in this study, suggests that the evidence supporting specific sleep factors as independent risk factors for myopia remains weak. Increased education (possibly linked to increases in near-work) and decreased time outdoors have been identified as the two major environmental risk factors for myopia (6–8, 43, 68). Sleep factors may serve as surrogate markers of either near-work, time outdoors, or both of these major risk factors for myopia. The extent to which later bedtime, shorter sleep or poorer sleep simply reflect longer duration spent on near-work or screen time (17, 65, 69, 70), or near-work activity close to bedtime (27) needs to be clarified. Further large prospective studies are required to evaluate if sleep factors are independently associated with myopia, with careful adjustments for established risk factors for myopia.

The strengths of this study include the capture of multiple sleep factors and the use of cycloplegic refraction data. The findings from this study should be interpreted considering the following limitations. First, the GUSTO myopia study was nested in the main GUSTO study assessing multiple childhood outcomes. Due to limits on the number of tests that could be performed at each study visit, sleep factors and myopia assessments of the subset GUSTO myopia study were limited to ages 8 and 9, respectively. As sleep factors and myopia were not assessed in the same year, changes to sleep patterns between ages 8 to 9 could not be precluded, although the likelihood of large changes within 1 year was likely to be low. In a meta-analysis of 9 studies (29,663 children) conducted in Asian, European, and Middle Eastern countries, the mean duration of sleep in children aged 8 years (mean: 9.3 [range: 7.8–10.8] h/day) was similar to that in children aged 9 years (mean: 9.3 [range: 7.8–10.8] h/day) (71). Second, the cross-sectional assessments do not allow for the capture of temporal patterns in sleep factors and ocular parameters. Third, for associations of the duration of sleep with SE or AL, the current sample size was adequately powered (at 80%) to detect effect sizes of 0.118 and 0.122, respectively, but not smaller effect sizes. Fourth, given the constraints of administering “gold standard” polysomnography (72), which are more disruptive and resource-intensive on a broad scale, subjective assessment of sleep was performed using questionnaires, which may be prone to recall bias. Although we expect the recall bias to be minimal given the questionnaire elicited parents' responses on their child's habitual sleep patterns during the “most recent typical week,” validated and objective measures of sleep should be considered where feasible to corroborate the subjective measurements. A recent study demonstrated that parental reports tend to over-estimate the duration of sleep (compared to polysomnography), however, the observed differences were small, with a substantial agreement between the two methods [intraclass correlation coefficient of 0.78, *p* < 0.01; (73)]. Validated objective measures (i.e., wrist-worn actigraphy) may provide information on unique sleep factors, over different timescales, and sampling resolution. However, as actigraphy may be limited in capturing certain aspects of sleep, such as wake after sleep onset (74), questionnaires may remain an indispensable instrument for assessing sleep disruptions or quality of sleep. Finally, although we reported null associations between specific sleep factors (which are closely regulated by the intrinsic circadian clock) and myopia, studies directly assessing circadian rhythms may be required to further elucidate potential circadian effects on myopia, independent of sleep factors.

In conclusion, our study results showed that sleep quality, duration, timing, and the consistency of specific sleep factors at age 8 were not independently associated with myopia, SE, or AL among school-aged children aged 9 years in Singapore. Although the current findings do not support associations of specific sleep factors with myopia, much larger longitudinal studies may be required to corroborate these results.

## Data Availability Statement

The datasets presented in this article are not readily available because the dataset can only be made available upon request and approval by the GUSTO Executive Committee. Requests to access the datasets should be directed to mijie@u.nus.edu.

## Ethics Statement

The studies involving human participants were reviewed and approved by National Healthcare Group Domain Specific Review Board (D/2009/021), Singhealth Centralized Institutional Review Board (2018/2767), and both the National Healthcare Group Domain Specific and Singhealth Centralized Institutional Review Boards (2018/2270; R1517/16/2018). Written informed consent to participate in this study was provided by the participants' legal guardian/next of kin.

## Author Contributions

ML: drafting of manuscript. ML, C-ST, LX, L-LF, ET, SC, MA, S-MS, and CS: conceptualization, design, analysis, or interpretation of data. ML, FY, C-HS, ET, SC, and S-MS: acquisition of data. S-MS: acquisition of funding. All authors were involved in the critical revision, review, and approval of the manuscript.

## Funding

This study was supported by the Agency for Science Technology and Research, Singapore (A^*^STAR) and JANSSEN World Without Disease Grant (JRBMRR151701) and the Singapore National Medical Research Council Grants (NMRC/TCR/012-NUHS/2014 and NMRC/TCR/004-NUS/2008). The funders were not involved in the study design, collection, analysis, interpretation of data, the drafting of the manuscript, or the decision to submit for publication.

## Conflict of Interest

The authors declare that the research was conducted in the absence of any commercial or financial relationships that could be construed as a potential conflict of interest.

## Publisher's Note

All claims expressed in this article are solely those of the authors and do not necessarily represent those of their affiliated organizations, or those of the publisher, the editors and the reviewers. Any product that may be evaluated in this article, or claim that may be made by its manufacturer, is not guaranteed or endorsed by the publisher.
